# Transcriptome-guided engineering of a native niacin transporter in *Lactiplantibacillus plantarum* unveils metabolic rewiring for NMN biosynthesis

**DOI:** 10.3389/fmicb.2025.1637666

**Published:** 2025-07-14

**Authors:** Linghui Kong, Xinyu Li, Qing He, Qingshou Yao, Lianzhong Ai, Jiayang Qin

**Affiliations:** ^1^School of Pharmacy, Binzhou Medical University, Yantai, Shandong, China; ^2^School of Agriculture and Biology, Shanghai Jiao Tong University, Shanghai, China

**Keywords:** nicotinamide mononucleotide, niacin transporter, multicopy engineering, transcriptomic analysis, CRISPR/Cas9, *Lactiplantibacillus plantarum*

## Abstract

**Introduction:**

β-nicotinamide mononucleotide (NMN), a precursor of NAD^+^, holds promise as a functional food ingredient for mitigating age-related decline. This study enhanced NMN biosynthesis in probiotic *Lactiplantibacillus plantarum*.

**Methods:**

A putative niacin transporter, *lp2514*, was identified via molecular docking and validated by CRISPR/Cas9. A dual-copy expression strategy was also employed to increase NMN production. In parallel, RNA-seq was used to analyze genome-wide transcriptional changes associated with enhanced NMN biosynthesis.

**Results:**

Overexpression of *lp2514* increased NMN production by 62.3%, and a dual-copy strategy raised NMN titers to 203 μmol L^−1^-269% increase compared to empty-vector control without NAM and the highest yield reported in lactic acid bacteria. Transcriptomic analysis revealed 598 differentially expressed genes, including upregulated ribosomal proteins (*rpsJ, rplE*) and NAD^+^ salvage enzymes (*aspA*), indicating enhanced translation and precursor flux. Deleting *cinA*, encoding a metabolic constraint, further boosted NMN levels, confirming transcriptomic predictions.

**Discussion:**

This combined transporter engineering and transcriptome-guided strategy establishes a food-grade *L. plantarum* platform for efficient NMN production in functional fermented foods.

## 1 Introduction

β-nicotinamide mononucleotide (NMN), one of an important precursor of nicotinamide adenine dinucleotide (NAD^+^), is naturally present as a bioactive nucleotide (Cheng et al., 2024; He et al., 2022). Given the decline in NAD^+^ levels with increasing age, NAD^+^ supplementation could offer potential benefits in preventing age-related diseases, including metabolic disorders and cardiovascular dysfunctions (Nadeeshani et al., 2022; Poddar et al., 2019, Kuerec et al., 2024; Yang et al., 2021; Yoshino et al., 2018). Owing to its beneficial effects, NMN has gained attention as a functional food ingredient for supporting healthy aging and disease prevention (Luo et al., 2023). While chemical synthesis and enzymatic methods for NMN production exist, microbial fermentation offers a sustainable and cost-effective alternative (Bi et al., 2023; Wang et al., 2023).

**Figure 1:**
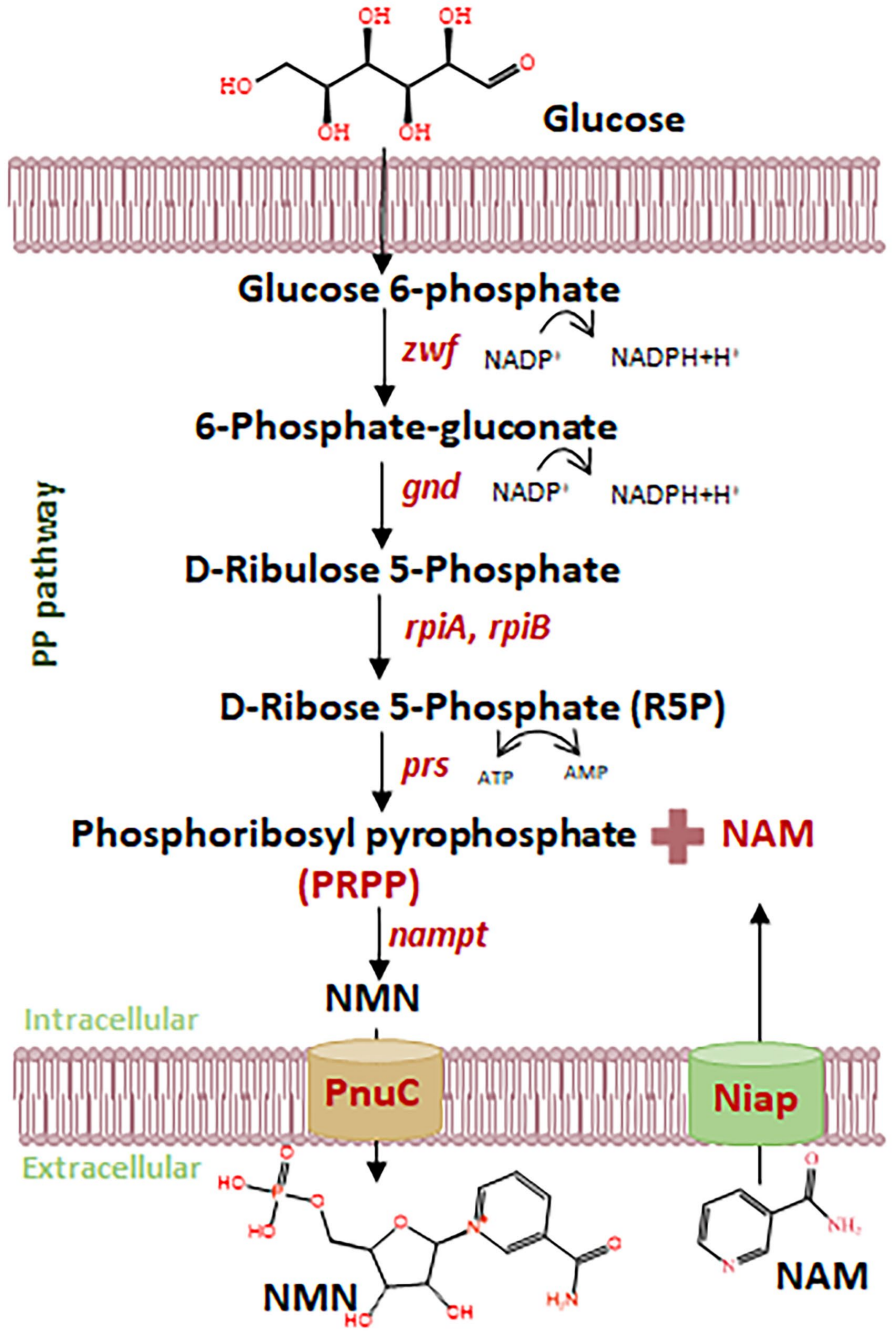
Schematic diagram of NMN biosynthesis using NAM and glucose. *Nampt*, NAM phosphoribosyltransferase; *prs*, ribose-phosphate diphosphokinase; *zwf*, glucose 6-phosphate dehydrogenase; *gnd*, 6-phosphogluconate dehydrogenase; *rpiA*, ribose 5-phosphate isomerase A; *rpiB*, ribose 5-phosphate isomerase B; NiaP, niacin transporter.

*Lactiplantibacillus plantarum*, a generally recognized as safe (GRAS) lactic acid bacterium (LAB), has emerged as an attractive microbial chassis for functional compound biosynthesis, due to its probiotic attributes, food compatibility, and robust metabolic versatility (Liu et al., 2017; Oleksy and Klewicka, 2016; Kong et al., 2024). Recent studies in *Escherichia coli* have demonstrated the feasibility of NMN production from inexpensive substrates such as nicotinamide (NAM), primarily through enhancement of NAM uptake and its conversion via the NAD^+^ salvage pathway (Huang et al., 2023; Liu et al., 2021; Maharjan et al., 2021; Shoji et al., 2021) (Figure 1). Marinescu et al. (2018) proposed a strategy for cost-effective NMN production in the presence of NAM, resulting in the production of NMN at 15.42 mg L^−1^. The yield of NMN was 16.2 g L^−1^ with a molar conversion rate of 97.0% from NAM in *E. coli* (Huang et al., 2022). These studies suggest that the biosynthesis of NMN by inexpensive NAM uptake has the advantage of reducing production costs. NAM often remains at low concentrations within the host, and niacin transporters (NiaPs) restrict the entry of NAM into the host cell (Bao et al., 2025). However, *E. coli* is not suitable for direct application in food systems, and efficient NAM transporters for NMN biosynthesis have not been characterized in LAB.

In this study, a native NiaP gene, *lp2514*, was identified and engineered in *L. plantarum* WCFS1-a well-characterized strain, with CRISPR-based tools to investigate its capacity for food-safe NMN production (Huang et al., 2022; Kleerebezem et al., 2003). We hypothesize that *lp2514* overexpression, combined with multicopy chromosomal integration, enhances NAM uptake and metabolic flux toward NMN, while transcriptomic profiling elucidates the systemic adaptations underlying yield optimization. This integrated approach establishes a food-grade microbial platform for functional NMN-enriched fermentation and provides new insights into metabolic control points for probiotic strain optimization.

## 2 Materials and methods

### 2.1 Bacterial strains, plasmids, and culture conditions

All strains and plasmids used in this study are listed in Table 1. *E. coli* DH5α strains were employed as the cloning hosts for plasmid construction. *E. coli* DH5α containing genome editing plasmid was cultivated in Luria-Bertani medium supplemented with 50 μg mL^−1^ kanamycin (Kan). *L. plantarum* WCFS1 was routinely cultured on MRS media without agitation. Then, 10 μg mL^−1^ erythromycin (Em) and 10 μg mL^−1^ chloramphenicol were added as needed. An appropriate quantity of NAM was introduced during the culture of *L. plantarum* WCFS1 to increase the biosynthesis of NMN.

**Table 1:** Strains and plasmids used in this study.

Strain/plasmid	Characteristics	Source
*Escherichia coli* DH5α	Host for cloning	Our lab
*Lactococcus lactis* NZ9000	Wild type; Cloning host for LLNZ00315 gene	Our lab
*Streptococcus thermophilus* S-3	Wild type; Cloning host for orf1015 gene	Our lab
*Lactobacillus plantarum* WCFS1	Wild type;	Our lab
WCFS1Δ*lp2514*	*lp2514* knockout mutant of WCFS1	This study
WCFS1Δ*cinA*	*cinA* knockout mutant of WCFS1	This study
pKLH32	Shuttle vector for *E. coli* and lactic acid bacteria with the constitutive P_23_ promoter; kanamycin resistance (Kan^r^)	Our lab
pKLH32-LLNZ00315	pKLH32 derived, carrying *LLNZ00315* gene from *L. lactis* at *Bam* HI and *Eco* RI sites; Kan^r^	This study
pKLH32-orf1015	pKLH32 derived, carrying *orf1015* gene from *S. thermophilus* at *Bam* HI and *Eco* RI sites; Kan^r^	This study
pLXY04	pKLH32 derived, carrying *lp2514* gene at *Bam* HI and *Eco* RI sites; Kan^r^	This study
pLXY09	Derived from pLXY04, for overexpression of *lp2514* gene (two copy gene of lp2514)	This study
pLXY10	Derived from pLXY09, for overexpression of *lp2514* gene (three copy gene of lp2514).	This study
pLCP	CRISPR/Cas9 system for *L. plantarum* erythromycin (Em^r^)	Huang et al. (2019)
pLH01	*L. plantarum* recombination helper plasmid, chloramphenicol (Cm^r^)	Huang et al. (2019)
pHH13	pLCP derived, carrying arms of *cinA* from WCFS1 and sgRNA at *Apa* I and *Xba* I sites; Kan^r^	This study
pLXY07	pLCP derived, carrying arms of *lp2514* from WCFS1 and sgRNA at *Apa* I and *Xba* I sites; Kan^r^	This study

### 2.2 Plasmid constructions

All primers used in this study are listed in Table 2, and the successful plasmids were validated by DNA sequencing. The pair of primers was used to amplify *LLNZ00315*, *orf1015* and *lp2514* genes from *L. lactis* NZ9000, *Streptococcus thermophilus* S-3 and WCFS1, respectively, and inserted into pKLH32 (digested with *Bam* HI/*Eco* RI) to create plasmids for NMN production. To further enhance *lp2514* expression, multi-copy expression cassettes were constructed. A second copy of *lp2514* was amplified and inserted into a separate expression region of the pKLH32 backbone, generating pLXY09 (dual-copy *lp2514*). To explore the effect of additional copies, pLXY10 was constructed by inserting three tandem copies of *lp2514*.

**Table 2:** Primers used in this study.

Primers	Sequence (5’-3’)
Lp2514-F	AATGACAATGATGTTGGATCCATGGTCGAAAGTAAAAGAAATAGTG
Lp2514-R	AGATCTCGAGCTCTAGAATTCTTAATCATCCGTCTCTTGATTCG
gRNA-lp2514-F	CATACTATGATATATTCTAGACCGCGCAACACCAAAACGCTGTTTTAGAGCTAGAAATA
gRNA-new-R	AAAAAAAGCACCGACTCGGT
lp2514-up-F	ACCGAGTCGGTGCTTTTTTTTCACGCCTGTACACCACGAAG
lp2514-up-R	GTATCGGTGGCAGATATAAATTAAATCG
lp2514-down-F	TTTATATCTGCCACCGATACTCCAATACTCCCCCAAATTTCCTA
lp2514-down-R	TCTTTTTCTAAACTAGGGCCCATCAAAGCGGATGTCTGGGAA
lp2514-confirm-F	GGCACAATGATGACACATGCC
lp2514-confirm-R	CAATGCGCGTTACCATTAATCGC
lp2514-inter-F	TGCCGGAATGCCAAATCCAA
lp2514-inter-R	GTGGTCCGCTAAATATCGACGAG
gRNA-CinA-F	catactatgatatattctagaaaccccggacacactgccgagttttagagctagaaata
CinA-up-F	accgagtcggtgctttttttCATTTTCACGGCCTTGACCA
CinA-up-R	ACGAACTTTTGTTCGCTTTTTGCTT
CinA-down-F	ctttaactccaaacttctaaGCCATATATGCCTCCCCGTA
CinA-down-R	tctttttctaaactagggcccACGACGGTTAAGTTCCAGTTTG
pLL-seq-F	CACTGATTGGTGTATCATTTCGT
pLL-seq-R	CATATCAAAGGGAAAACTGTCCA
CinA -confirm-F	CTCCGTCATGACATCAGGATGC
CinA -confirm-R	AACGCTAAAGGCGGATGGC
CinA -inter-F	CAACATCAGTATTTAGCTGCTGCTTAG
CinA -inter-R	CAGGTCCACCCCGTGAGTTA
Lp2514-real-R	CCTGCAGTTAGCTGCCAACTTTCTT
Lp1032-real-F	AGCTGACAAGATTGTCGAAACGG
Lp1032-real-R	CACGTGAGTCCTTAAACTTATGTGGT
Lp1047-real-F	GTAATGCAAGTGCCTAAGCTGGC
Lp1047-real-R	CTTCAACTGCTTCGTCTAAGTTTTTAGC
Lp1047-real-F	TCGACCGGAGATATCTTCCGT
Lp1047-real-R	GTGACTTCGTCTGGTACTAAGTTACCT
Lp2900-real-F	CGTCACAACAAGCCATAGATTGTAT
Lp2900-real-R	GCGTTCACATCTAAGTTTGTTGCT
Lp2710-real-F	TTATTGATTGGCGCGGCC
Lp2710-real-R	AACGTTGCGATCCCACACA
Lp2830-real-F	ACGTGGCTGAGTTAGTCCG
Lp2830-real-R	CCTGAATTATTACATCGACGTCGCT
Lp2771-real-F	GTTCCTGACAATGGTAGCTTTGTCA
Lp2771-real-R	GACGGAAGTAGTCGAGGTCAC
Lp3556-real-F	GCTGCTTCAACTGGTAAGCCAG
Lp3556-real-R	GAGCCAGAAAGTATGACGTTGAGTCC
Lp3545-real-F	TGCATCTTGGTTAATAGCACCCTTG
Lp3545-real-R	ACGTATCGTGGCTCACGG
Lp0230-real-F	ACGAATGCAACCCAAAGCG
Lp0230-real-R	CGGCATAACCATATTACTTAGTGCAC
Lp2302-real-F	GGTACTGCTTGGCCTTCAC
Lp2302-real-R	GGTATCAGTTTTACAGGCGTTGC
Lp3555-real-F	GACCATAATAGGCACTTGCACCA
Lp3555-real-R	ATACTGCGCAATTGACCCGT
Lp2183-real-F	GGCATCCTGAATCTGACCAGT
Lp2183-real-R	TGGTTGCAACGGCATTCC
Lp2703-real-F	CGCCACCATTGCCATCC
Lp2703-real-R	ATGCGATTATCATGCATCCGGC
Lp2251-real-F	TGGCTTCCTTAGTTGGAATCGATG
Lp2251-real-R	TGCACTCAGTTCGGTCTTAATGC
Ter-P23-F	GAGACGGATGATTAAGAATTCAGTGATTAGTCAAAGAATGGTGATGACA
Ter-P23-R	AGCGAAGCGAACACTTGATTTTT
p23-lp2514-F	AATCAAGTGTTCGCTTCGCTCGAAAAGCCCTGACAACCCT
p23-lp2514-R	ATCGATAGATCTCGAGCTCTATTAATCATCCGTCTCTTGATTCGG
orf1015-F	AATGACAATGATGTTGGATCCTTGAAAATAAAAAAATATGCAGCAT
orf1015-R	AGATCTCGAGCTCTAGAATTCTTACTTATCAATGCTTAGTT
LLNZ00315-F	AATGACAATGATGTTGGATCCATGATTAAACAATTTTTAGGTATTATTAACTCAG
LLNZ00315-R	AGATCTCGAGCTCTAGAATTCTTAATTCTTTTTATCTACCAAATCTAAAAGGA
p23-lp2514-R1	ACCATTCTTTGACTAATCACTTTAATCATCCGTCTCTTGATTCGG

The homologous arms of *lp2514* were amplified from WCFS1. The specific guide RNA (gRNA), composed of a 20 bp protospacer sequence, was cloned from pLCP using gRNA-lp2514-F/gRNA-lp2514-R primers (Huang et al., 2019). The plasmid pLCP (CRISPR/Cas9 system) was digested with the restriction enzymes *Apa* I and *Xba* I, and then ligated to the fragments using the ClonExpress one-step cloning kit, resulting in the creation of recombinant plasmid pLXY07 for *lp2514* gene knockout. The pHH13 plasmid was constructed as described above.

### 2.3 Genome manipulation procedures using CRISPR/Cas9 in *Lactiplantibacillus plantarum*

The competent cells of *L. plantarum* WCFS1 were generated in accordance with previous report (Huang et al., 2019). The helper plasmid pLH01 was transformed into *L. plantarum* WCFS1, and the resulting positive transformants were screened on Cm plates for the preparation of the competent cells. The plasmid pLXY07 was subsequently transferred into WCFS1 via electroporation, and cultivated for 72 h at 37°C. To confirm positive recombinants, colony PCR analysis was performed using the primers (lp2514-confirm-F/lp2514-confirm-R; lp2514-inter -F/lp2514-inter -R) that targeted the homologous regions of the *lp2514* gene. After successful genome editing, the recombinant strain was serially passaged without antibiotics for 2–3 generations to facilitate plasmid curing. Colonies were screened for the loss of antibiotic resistance, and plasmid loss was further confirmed by colony PCR.

### 2.4 Fermentation of engineered *Lactiplantibacillus plantarum* for *lp2514* expression and NMN production

The plasmids pLXY04, pLXY09, pLXY10 and pKLH32 (as a control) were introduced into *L. plantarum* WCFS1. The engineered *L. plantarum* WCFS1 and WCFS1Δ*lp2514* strains were grown at 37°C. Cell growth was quantified by measuring optical density at 600 nm (OD 600 nm) with a UV–Vis spectrophotometer. The fermentation broths were collected by centrifugation (12,000 *g*, 10 min, 4°C), and the cells were resuspended in buffer solution for disruption using a low temperature ultrahigh-pressure continuous flow cell disrupter. The resulting cell lysate was then centrifuged (12,000 *g*, 3 min, 4°C), and the supernatants were decanted for the NMN assay. NMN was derived from *L. plantarum* and detected with reported fluorometric methods (Kong et al., 2023; Marinescu et al., 2018).

### 2.5 Real-time quantitative PCR (RT-qPCR) assay

Total RNA was extracted from *L. plantarum* WCFS1/pKLH32, WCFS1/pLXY04, WCFS1/pLXY09, and WCFS1/pLXY10 using the RNAiso Plus reagent (TaKaRa, Japan) following the manufacturer’s protocol. First-strand cDNA was synthesized from 1 μg of total RNA using a reverse transcription kit. GAPDH was used as the internal control, and relative transcript levels were calculated using the 2^^−ΔΔCt^ method.

### 2.6 RNA-sequencing and transcriptomic analysis

Total RNA was extracted from *L. plantarum* strains WCFS1/pLXY04 (*lp2514* overexpression with 0.1% NAM) and WCFS1/pKLH32 (empty vector control with 0.1% NAM) using RNAiso Plus reagent (TaKaRa, Japan) following the manufacturer’s instructions. RNA purity and concentration were assessed using a NanoDrop spectrophotometer (Thermo Fisher Scientific, United States), and RNA integrity was verified via agarose gel electrophoresis and an Agilent 2,100 Bioanalyzer (Agilent Technologies, United States). Sequencing was conducted on an Illumina HiSeq 2000 platform (Majorbio Bio-Pharm Technology Co., Ltd., Shanghai, China). Clean reads were mapped to the reference genome of *L. plantarum* WCFS1, and gene expression levels were quantified as fragments per kilobase of transcript per million mapped reads (FPKM). Differentially expressed genes (DEGs) between the two groups (N04 vs. N32) were identified using DESeq2 software with thresholds of |log₂Fold Change| ≥ 1 and adjusted *p*-value (FDR) < 0.05. Gene Ontology (GO) and Kyoto Encyclopedia of Genes and Genomes (KEGG) enrichment analyses were performed to investigate functional categories and metabolic pathways significantly affected by *lp2514* overexpression.

### 2.7 Statistical analysis

All the experimental data were independently repeated in triplicate. Statistical analyses of the data were performed with GraphPad Prism and are presented as the means ± standard deviations (*p < 0.05 and **p < 0.01).

## 3 Results and discussion

### 3.1 Screening NAM transporter for NMN biosynthesis from LAB

**Figure 2:**
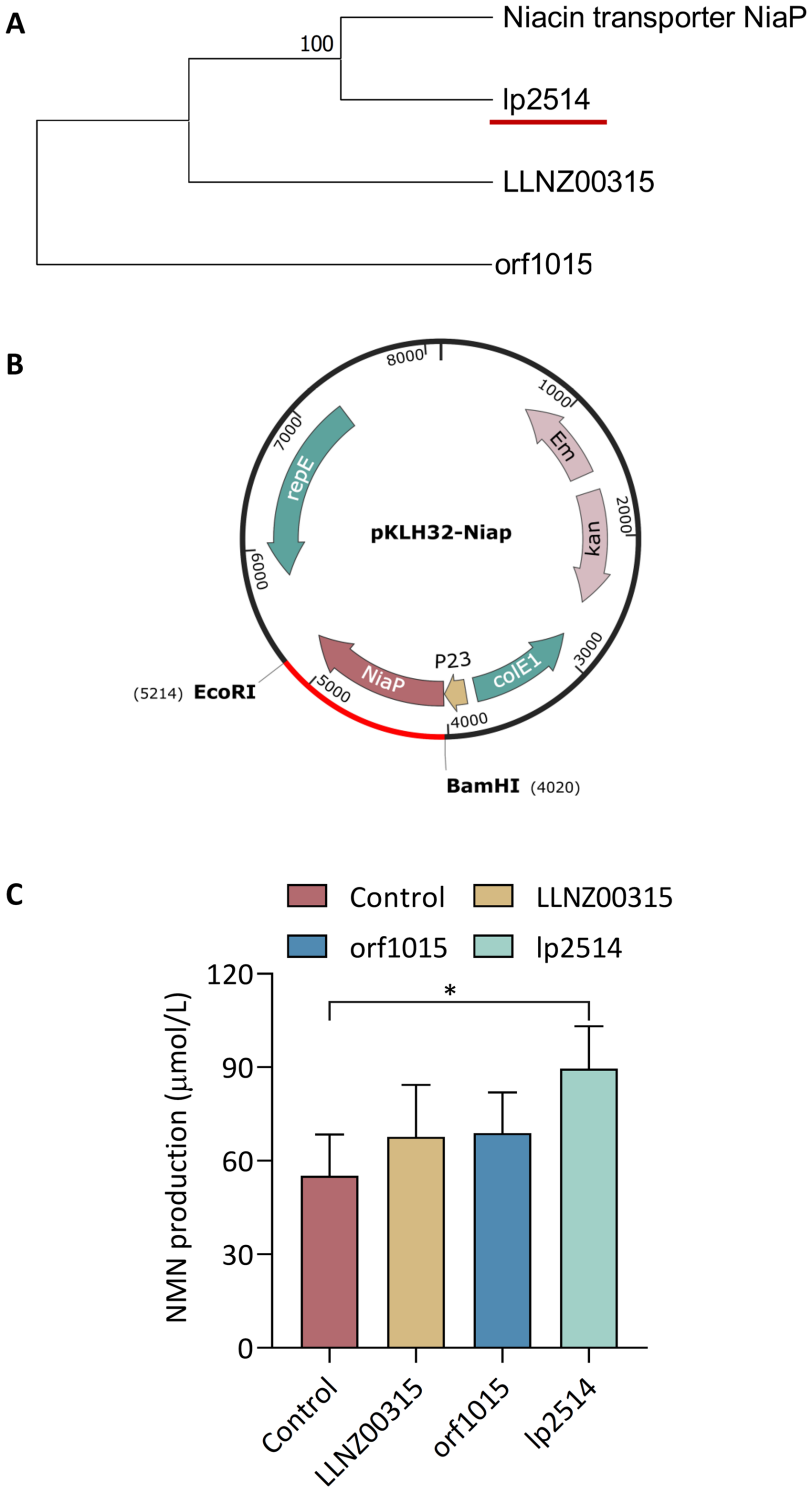
Screening of niacin transporters for NMN production in lactic acid bacteria. Phylogenetic analysis of NAM transporters in LAB **(A)**. Schematic representation of recombinant expression plasmids containing orf1015 from *S. thermophilus* S-3, LLNZ00315 from *L. lactis* NZ9000 and *lp2514* from WCFS1 **(B)**. The ability of different recombinant strains expressing NiaP to synthesize NMN **(C)**. *p < 0.05 , **p < 0.01.

The efficient biosynthesis of NMN by LAB using NAM as the substrate was shown to be an effective synthesis strategy in our previous study (Kong et al., 2023). The translocation of the NAM substrate into the host cell to increase NMN production requires a robust NAM transporter (Figure 1). However, there have been no reports on the endogenous proteins responsible for transporting NAM in LAB. Native proteins are more favorable for expression within the host. NiaP from *Burkholderia cenocepacia* significantly improved NAM uptake leading to an enhance in the synthesis of NMN from 185 mg L^−1^ to 231 mg L^−1^ (Shoji et al., 2021). *In silico* analysis revealed the presence of putative NAM transporters in LAB, including *L. plantarum, L. lactis and S. thermophilus.* Using BCNiaP as a query sequence for BLAST analysis with the target genomes, the genes encoding *orf1015* from *S. thermophilus* S-3, *LLNZ00315* from *L. lactis* NZ9000 and *lp2514* from *L. plantarum* WCFS1 were identified. Phylogenetic tree analysis showed that the NiaP from different sources clustered into distinct branches, and that *lp2514* from *L. plantarum* WCFS1 was positioned in close proximity to BCNiaP (Figure 2A). The above genes were subsequently inserted into the pKLH32 plasmid, and the resulting plasmids were transferred to *L. plantarum* WCFS1 (Figure 2B). Among these strains, strain WCFS1/pLXY04, which expressed *lp2514* could biosynthesize 89.6 μmol L^−1^ of NMN intracellularly, resulting in 62.3% increase in NMN levels, compared with those of strain WCFS1/pKLH32, which contained only the empty plasmid of pKLH32 (Figure 2C). These results clearly demonstrate that the expression of NiaP (*lp2514*) facilitates the production of NMN in *L. plantarum*, which is consistent with the phylogenetic tree. The *lp2514* gene markedly affected the NMN yield and was applied for subsequent experiments.

### 3.2 Identification of the *lp2514* gene via *in silico* molecular docking and CRISPR/Cas9 technology

**Figure 3:**
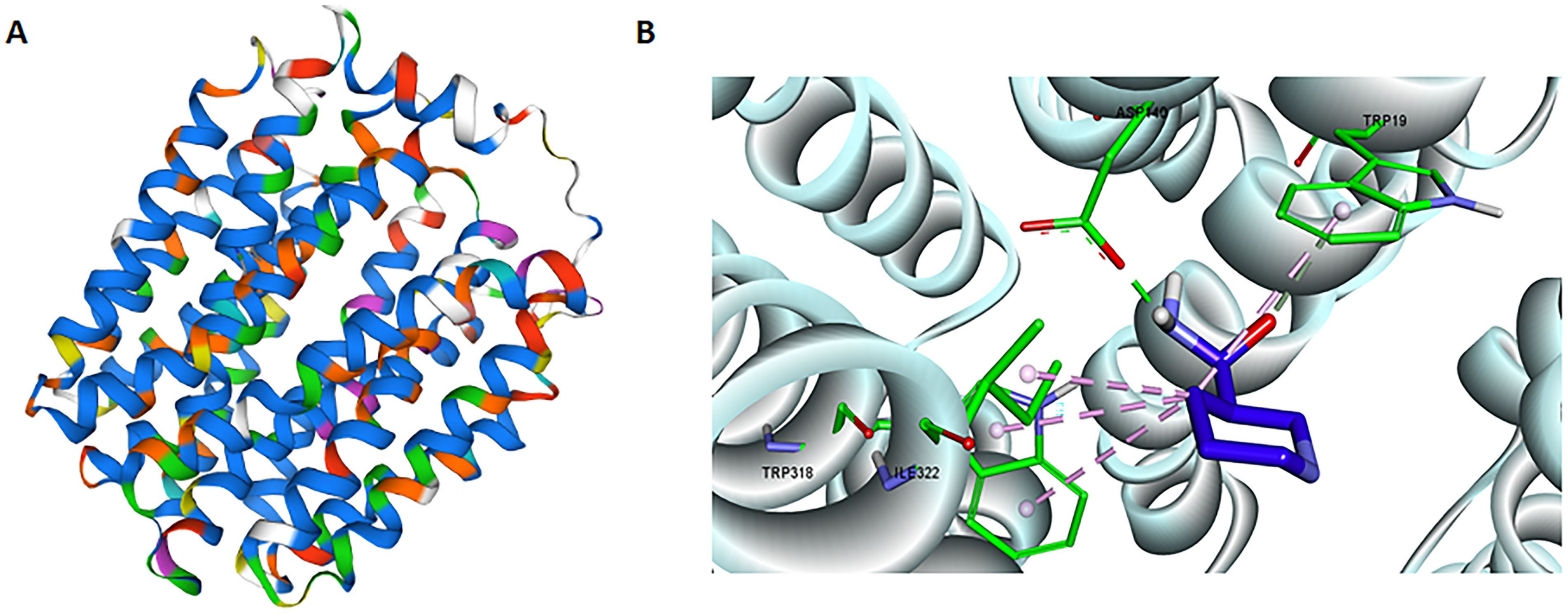
Structural model of *lp2514* and the key residues close to NAM. *In silico* tertiary structure modeling results for the lp2514 protein using the SWISS-MODEL server **(A)**. *In silico* molecular docking of NAM to lp2514 was simulated by docking server, and the residues (ASP140, TRP19, TRP318, and ILE322) are shown in stick format **(B)**.

We performed domain analysis of the NAM transporter using the search tool for conserved domains from the NCBI, and reported that BCNiaP and *lp2514* possessed the same domains as MFS_SV2_like, UhpC and MFS_1, suggesting that *lp2514* has the potential to conjugate NAM to NMN. The MFS transporter (UniProt A0A2R3JML5; seq similarity 53%; coverage 98%) was selected as template for homology modeling of *lp2514* (Figure 3A). The Ramachandran plot revealed that all the residues were predominantly in the most favored region (96.4%) and in an additional allowable area (3.6%), suggesting that the conformation of the generated model adheres to principles of stereochemistry (Supplementary Figure S1). The structural models of *lp2514* could be applied to simulate molecular docking with the substrate NAM molecule being docked in the substrate binding pocket of *lp2514* using a docking server. The key residues of ASP140, TRP19, TRP318, and ILE322 were located close to the substrate NAM (Figure 3B). These findings suggest that the lp2514 protein has strong binding affinity for the NAM substrate.

**Figure 4:**
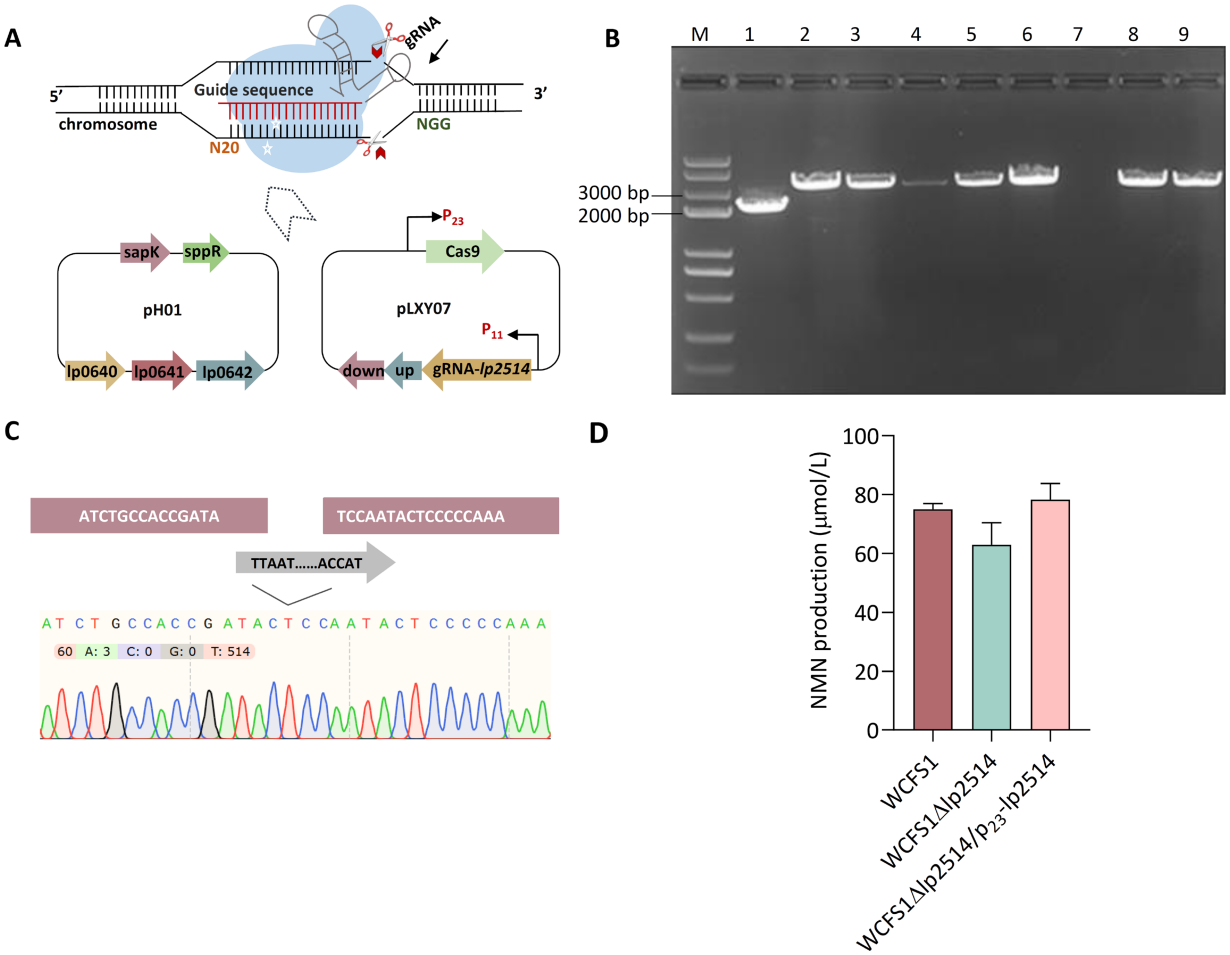
Characterization of the effects of *lp2514* on NMN biosynthesis via CRISPR/Cas9 in *L. plantarum* WCFS1. Schematic representation of the pH01 and pLXY07 plasmids used for *lp2514* gene knockout **(A)**. Identification of *lp2514* deletion using colony PCR **(B)**. The wild-type WCFS1 was used as control. The 2.1 and 3.3-kb bands indicate the positive and negative mutants, respectively. 1–7: transformants; 8–9: Wild-type. Sequencing validation of the WCFS1Δ*lp2514* mutant **(C)**. Intracellular NMN production of WCFS1Δ*lp2514* mutant and WCFS1Δ*lp2514/p23-lp2514* strains **(D)**.

Considering the potential role of the *lp2514* gene in promoting NMN biosynthesis via NAM uptake, it was worthwhile to verify its functionality by knockout of *lp2514* via CRISPR/Cas9 technology in *L. plantarum* WCFS1 (Figure 4A). A pLXY07 plasmid was constructed to develop the gene deletion mutant of *lp2514*, which was subsequently delivered into WCFS1 (Supplementary Figure S2). One positive mutant (WCFS1Δ*lp2514*) was successfully generated from eight transformants via colony PCR (Figure 4B). The acquisition of a deletion was verified by sequencing the PCR amplification product of the *lp2514* mutant (Figure 4C). When 1 g L^−1^ NAM was added, the intracellular NMN production of WCFS1Δ*lp2514* was decreased by 17% compared with that of the wild-type WCFS1 (Figure 4D). Interestingly, when *lp2514* was overexpressed in the WCFS1Δ*lp2514* strain, NMN production was restored. These findings revealed that the *lp2514* gene plays a role as NAM transporter affecting the biosynthesis of NMN, which agrees well with our above results. This is the first report on newly developed NAM transporter screening for NMN biosynthesis in LAB.

### 3.3 Effect of *lp2514* on NMN biosynthesis via NAM uptake in *Lactiplantibacillus plantarum*

**Figure 5:**
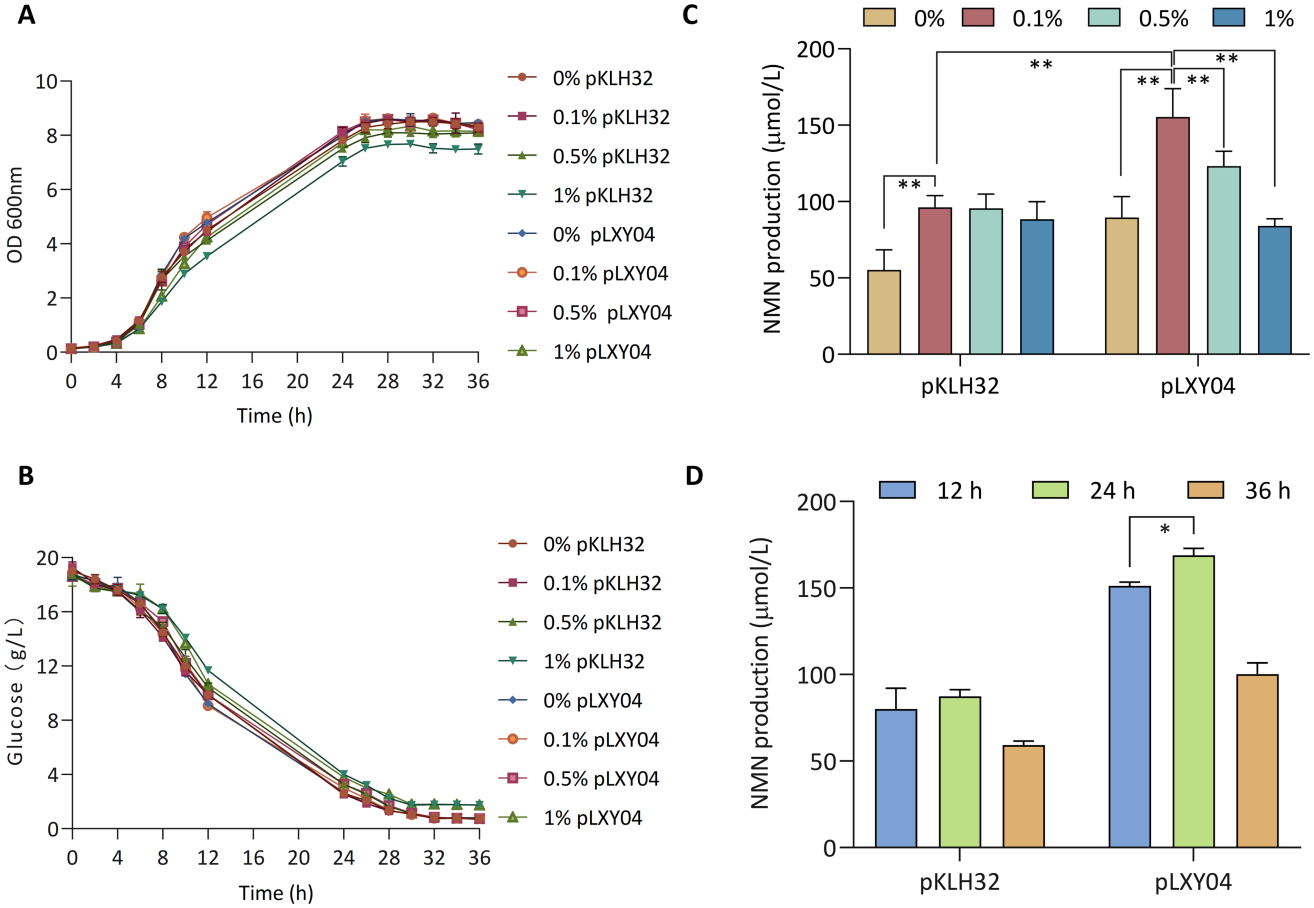
Effects of the NMN transporter *lp2514* on NMN biosynthesis. The growth curves (OD 600 nm) **(A)** and remaining glucose **(B)** of *lp2514* gene expression recombinants in MRS media supplemented with 0, 1, 5 and 10 g L^−1^ NAM. Effect of NAM concentration (0, 1, 5 and 10 g L^−1^) on NMN production by recombinant *L. plantarum* WCFS1/pLXY04 **(C)**. Effects of the different fermentation time (12, 24, and 36 h) on NMN production in MRS media supplemented with 1 g L^−1^ NAM **(D)**. *p < 0.05 , **p < 0.01.

To evaluate whether WCFS1/pLXY04 could increase in NMN production, various substrate concentrations of NAM and fermentation durations were tested. Overexpression of *lp2514* did not affect the growth of the recombinant strains WCFS1/pKLH32 and WCFS1/pLXY04 (Figure 5A). Kong et al. (2023) reported that various NAM concentrations had an impact on the growth of *L. lactis* NZ9000, under high concentration conditions (50 g L^−1^), NAM inhibited strain growth. The utilization of 50 g L^−1^ NAM exhibited toxicity toward recombinant *E. coli* (Marinescu et al., 2018). The growth of WCFS1/pKLH32 and WCFS1/pLXY04 was not limited by the addition of 1 g L^−1^ or 5 g L^−1^ NAM, although a slight decrease in the growth rate was observed with 10 g L^−1^ NAM. This finding is consistent with the findings of our previous study (Kong et al., 2023). Moreover, the growth rates are closely associated with the consumption of glucose (Figure 5B). NAM addition is a limiting factor for NMN biosynthesis, and various researchers have added different doses of NAM when is utilized NiaP (Jeanguenin et al., 2012; Shoji et al., 2021; Su et al., 2024). Compared with the control WCFS1/pKLH32, WCFS1/pLXY04 achieved significant NMN production, where varying amounts of NAM (1, 5 and 10 g L^−1^) were supplied to the MRS medium (Figure 5C). The maximum intracellular NMN titer of WCFS1/pLXY04 (155.5 μmol L^−1^; 1 g L^−1^ NAM) was synthesized after 12 h of fermentation, which was 182.7% greater than that of the control (55 μmol L^−1^) without NAM supplementation. Marinescu et al. (2018) reported that the addition of 1 g L^−1^ NAM resulted in the greatest NMN accumulation, which is consistent with our findings. These findings revealed that relatively high concentrations of NAM may not be conducive to NMN accumulation.

To determine the appropriate fermentation time for enhanced NMN production, the effects of fermentation for various time periods (12, 24 and 36 h) on NMN biosynthesis were evaluated, and the results indicated that fermentation times of 24 h (168.9 μmol L^−1^) or longer were was suitable for the biosynthesis of NMN (Figure 5D). Sugiyama et al. (2021) reported that *Fructobacillus tropaeoli* RD012354 presented the highest NMN production after 24 h cultivation. These findings revealed that the recombinant strain WCFS1/pLXY04, when cultured under optimized conditions (1 g L^−1^ NAM and 24 h cultivation), resulted in a 207% increase in the NMN yield, reaching 168.9 μmol L^−1^.

### 3.4 Multi-copy expression of *lp2514* unlock high-efficiency NMN biosynthesis in *Lactiplantibacillus plantarum*

**Figure 6:**
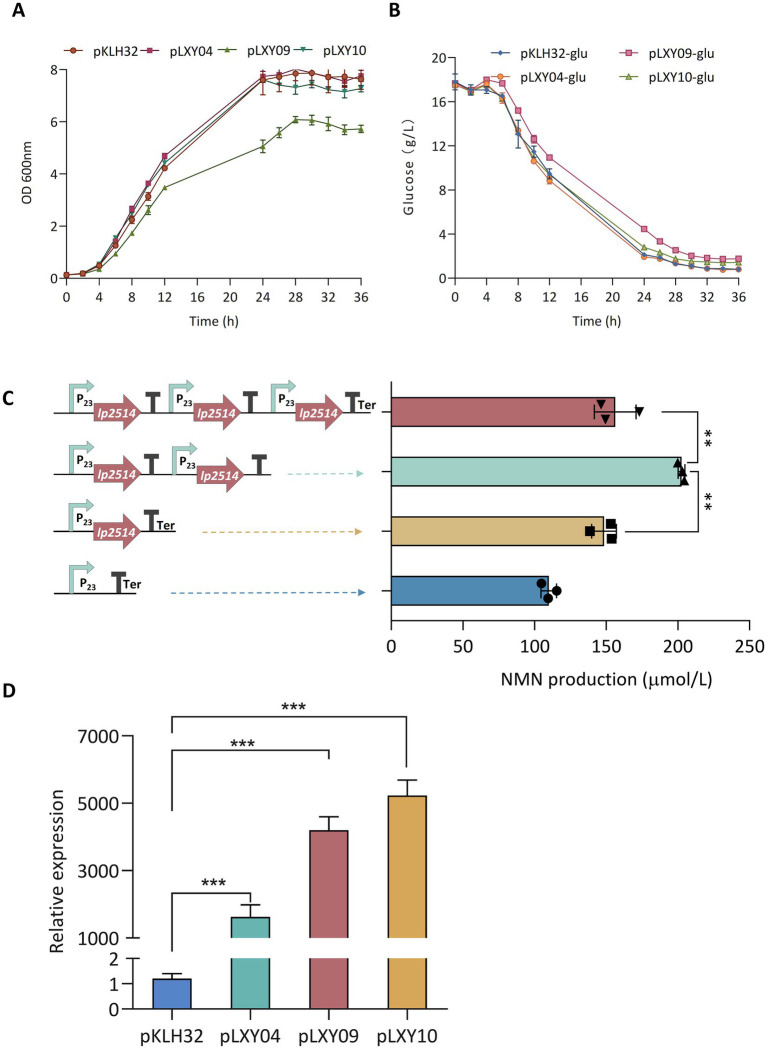
Expression of multicopy *lp2514* in *L. plantarum* WCFS1. The growth curves (OD600 nm) **(A)** and remaining glucose **(B)** of multicopy-lp2514 gene expression recombinants supplemented with 1 g L^−1^ NAM. Construction of plasmids with increasing copy numbers of the *lp2514* expression cassette, and analysis of the production of NMN in recombinants **(C)**. Analysis of the expression level by RT-qPCR in *L. plantarum* WCFS1 recombinants **(D)**. **p < 0.01 , ***p < 0.0001.

The multicopy expression cassette strategy represents a robust approach to increase natural product yields by increasing recombinant protein abundance, albeit with potential metabolic trade-offs. Cheng et al. (2023) employed a multicopy integration strategy to increase the production of germacrene A, which resulted in a 7.7-fold increase over the control. An optimal number of gene copies should be carefully studied because of the metabolic pressure associated with protein overexpression. To enhance the expression of *lp2514*, multicopy expression plasmids were constructed based on the pKLH32 vector. The *lp2514* gene was amplified using overlap extension PCR and inserted sequentially into the *Bam HI*/*Eco* RI sites of pKLH32 to generate plasmids harboring one (pLXY04), two (pLXY09), and three (pLXY10) tandem copies of *lp2514* under the control of the same promoter (Figure 6). Compared with that of WCFS1/pKLH32, the OD_600_ value of WCFS1/pLXY09 significantly decreased during 36 h cultivation, accompanied by a simultaneous reduction in the rate of glucose consumption (Figures 6A,B). The expression of multiple *lp2514* imposed a certain burden on the growth of the strain. Notably, an obviously greater quantity of NMN (203 μmol L^−1^) was substantiated by WCFS1/pLXY09 expressing two copies of *lp2514*, which increased by 37 and 85% compared with those of WCFS1/pKLXY04 and WCFS1/pKLH32, respectively (Figure 6C). Kong et al. (2020) reported that dual-copy families of the SOD, not four-copy families, presented the greatest activity, with a similar conclusion. The NMN production of WCFS1/pLXY10 also increased by 42% compared with that of the control strain (WCFS1/pKLH32), but slightly decreased compared with that of WCFS1/pLXY09. It is possible that *lp2514* overexpression in multiple copies may cause metabolic stress, which subsequently affects NMN biosynthesis. Compared with that of the control WCFS1/pKLH32 without NAM, the final NMN titer was increased by 269% via a series of regulatory mechanisms. The accumulation of NMN in WCFS1/pLXY09 was approximately 33.9-fold greater than that in *Fructobacillus durionis* RD011727 (Table 3). Moreover, the transcriptional analysis revealed a significant increase in the expression of *lp2514* in multicopy recombinants, exceeding that of the control strain by more than 1,000-fold (Figure 6D). The gene copy number is strongly correlated with the transcriptional level of *lp2514*, rather than with NMN production. This achievement represents the highest reported yield of NMN using NAM as substrate in *L. plantarum*, marking a significant advancement in the field of NMN biosynthesis by LAB. Although the yield of NMN synthesized has reached g L^−1^ levels (17.2 g L^−1^) by *E. coli*, which are much greater than those of LAB (Maharjan et al., 2023), our *L. plantarum* platform retains distinct benefits for functional-food applications. Specifically, *L. plantarum* has GRAS status, an intrinsic probiotic effect (supporting gut health and immune modulation), and the ability to perform food-grade fermentations without extensive downstream purification. These properties make LAB particularly well suited for developing NMN – enriched fermented products that can be marketed directly to health – conscious consumers. This study establishes *lp2514* multicopy engineering as a transformative strategy for LAB-based NMN bioproduction, bridging synthetic biology with functional food innovation.

**Table 3:** Comparison of NMN titer levels of different lactic acid bacteria strains.

Strains	NMN titer	References
*Fructobacillus tropaeoli* RD012353	1.5 mg L^−1^	Sugiyama et al. (2021)
*Fructobacillus tropaeoli* RD012354	1 mg L^−1^	Sugiyama et al. (2021)
*Fructobacillus durionis* RD011727	2 mg L^−1^	Sugiyama et al. (2021)
*L. plantarum* WCFS1	203 μmol L^−1^ (67.8 mg L^−1^)	This study

### 3.5 Transcriptomic analysis of NMN biosynthesis pathways in *lp2514*-overexpressing *Lactiplantibacillus plantarum*

#### 3.5.1 Screening of DEGs

**Figure 7:**
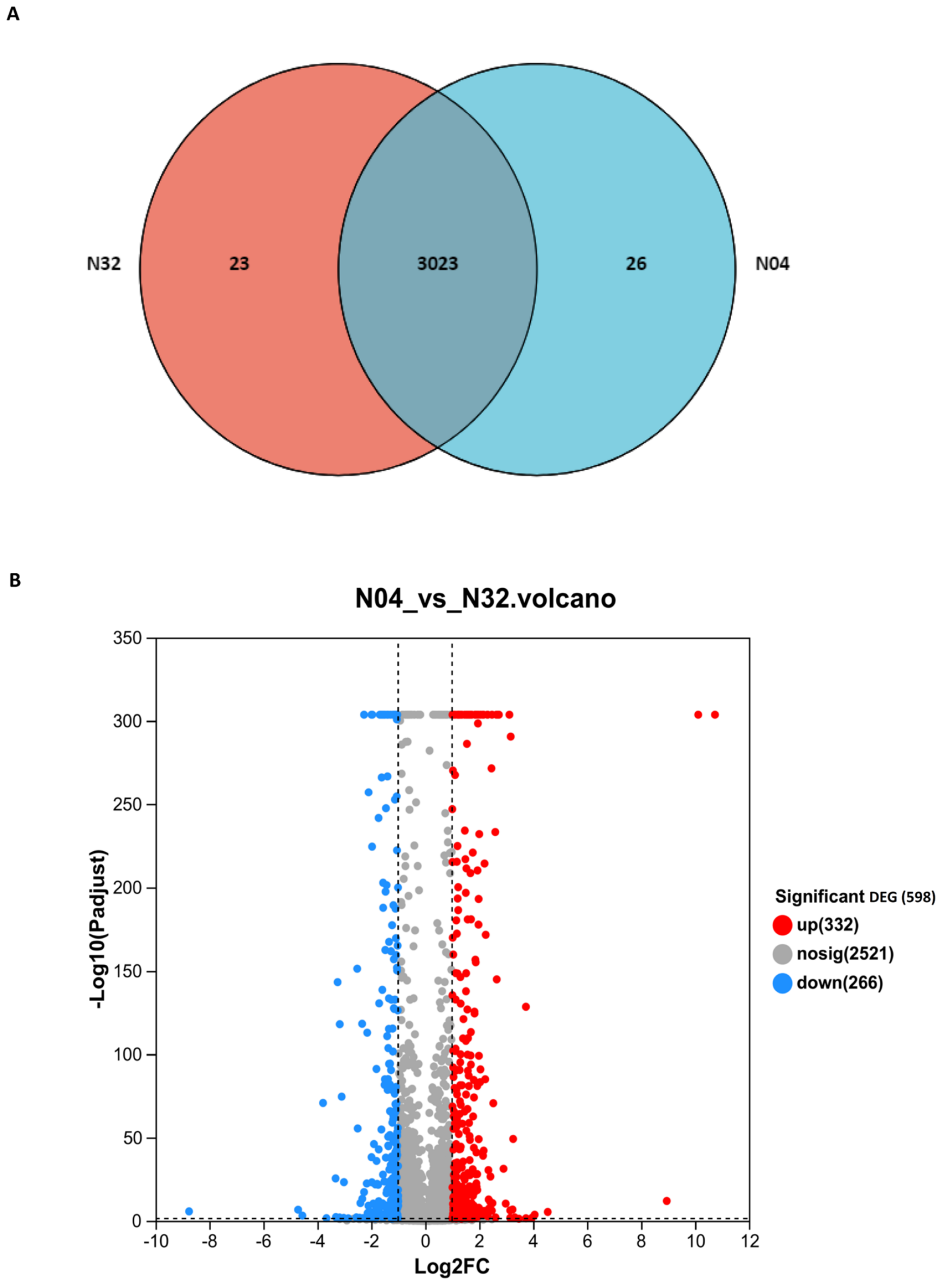
Transcriptomic profiling of *lp2514*-overexpressing *L. plantarum* (N04) versus control (N32). Venn diagram illustrating unique and shared genes between N04 and N32 **(A)**. Volcano plot of differentially expressed genes (DEGs). Significantly upregulated (332 genes, red; |log2FC| >1, FDR <0.05) and downregulated (266 genes, blue) genes are highlighted **(B)**.

To elucidate the transcriptional response of *L. plantarum* to *lp2514* overexpression in the presence of NAM, we conducted RNA-seq analysis comparing strain N04 (overexpressing *lp2514*) with N32 (empty vector control) (Figure 7). The Venn diagram highlights 29 unique DEGs in N04, suggesting specific regulatory targets of *lp2514* (Figure 7A). The DEGs are likely directly related to *lp2514* overexpression, further supporting its pivotal role in NMN biosynthesis. Volcano plot analysis revealed that 332 genes were significantly upregulated and that 266 genes were downregulated (|log2FC| > 1, FDR < 0.05) in N04 (Figure 7B). Each point represents a gene, with red indicating upregulation, blue indicating downregulation, and gray denoting no significant expression change. The upregulated genes are likely associated with NMN biosynthesis pathways, whereas the downregulated genes may pertain to competing metabolic pathways.

#### 3.5.2 GO analysis reveals functional rewiring toward NMN biosynthesis

**Figure 8:**
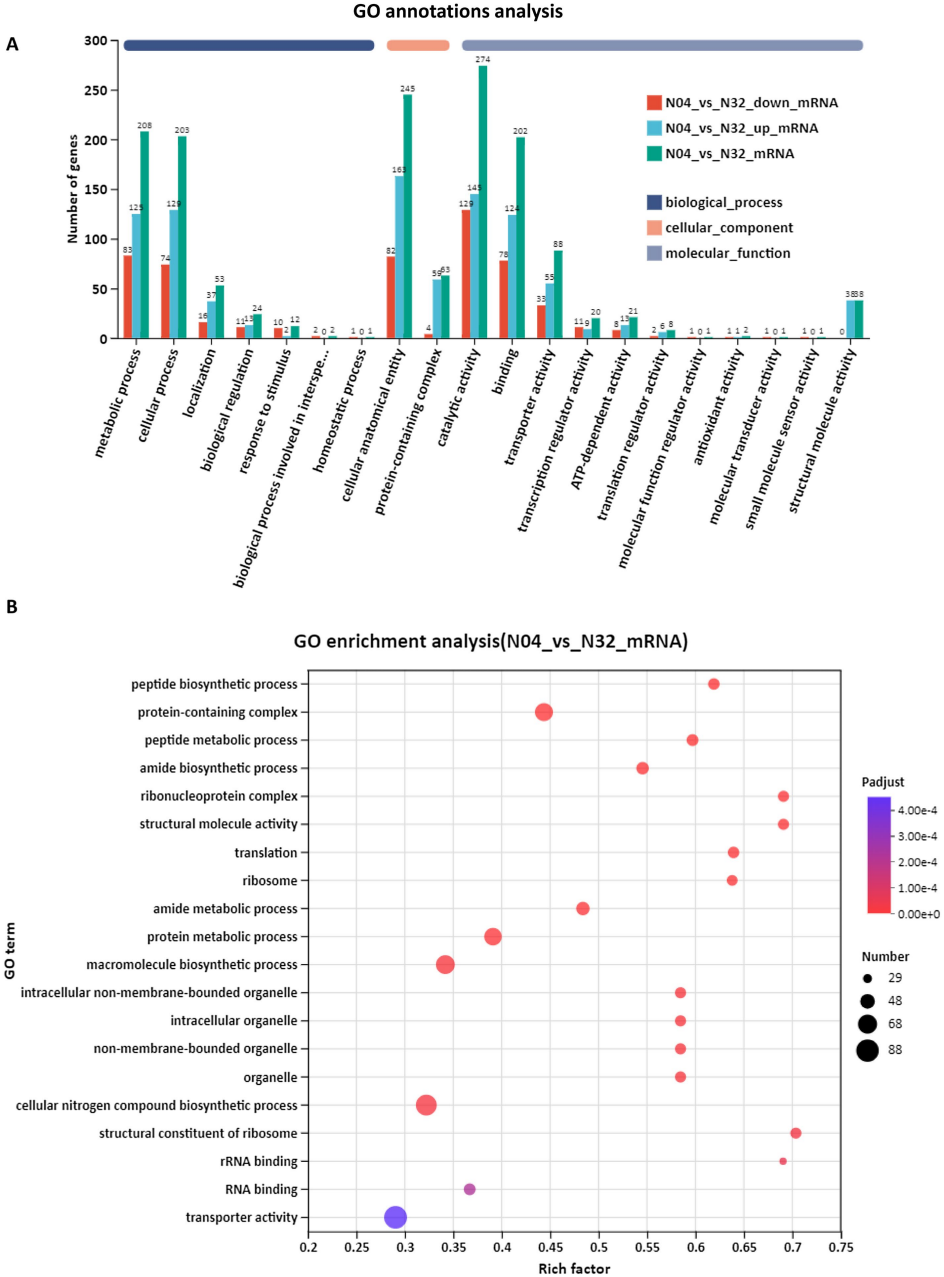
Gene Ontology (GO) annotation and enrichment analysis of N04 vs. N32. GO annotation summary of differentially expressed genes (DEGs) between N04 and N32 **(A)**. GO enrichment bubble plot showing significantly overrepresented GO terms in N04 vs. N32 **(B)**.

GO annotation revealed that the overexpression of *lp2514* in the N04 strain significantly alters the representation of gene categories compared to the N32 strain (Figure 8A). Notably, there is an increase in the number of DEGs associated with “transporter activity” and “catalytic activity” in N04, suggesting an enhanced capacity for the absorption of NAM and its conversion into NMN. Furthermore, elevated counts in categories “translation” and “structural constituent of ribosome” suggest a system-wide enhancement of the protein synthesis machinery, which is consistent with an increased demand for NMN-biosynthetic enzymes. GO enrichment analysis (represented as a bubble plot) identifies specific processes that were most strongly upregulated in N04 (Figure 8B). “Transporter activity” had the lowest adjusted *p*-value and a moderately rich factor, underscoring *lp2514*’s direct role in NAM import. There was a marked overrepresentation of biological processes related to peptide biosynthesis (rich factor = 0.65) and ribosome assembly (rich factor = 0.7) in N04 compared with N32. This is consistent with the 332 upregulated genes identified in the volcano plot, many of which encode ribosomal proteins, such as *rpsJ* and *rplE*. The enrichment of ribosome-associated terms indicates a potential connection to the efficient expression of enzymes required for NMN biosynthesis, while the increased activity of structural molecules may offer metabolic support to the cell.

#### 3.5.3 KEGG analysis reveals functional rewiring toward NMN biosynthesis

**Figure 9:**
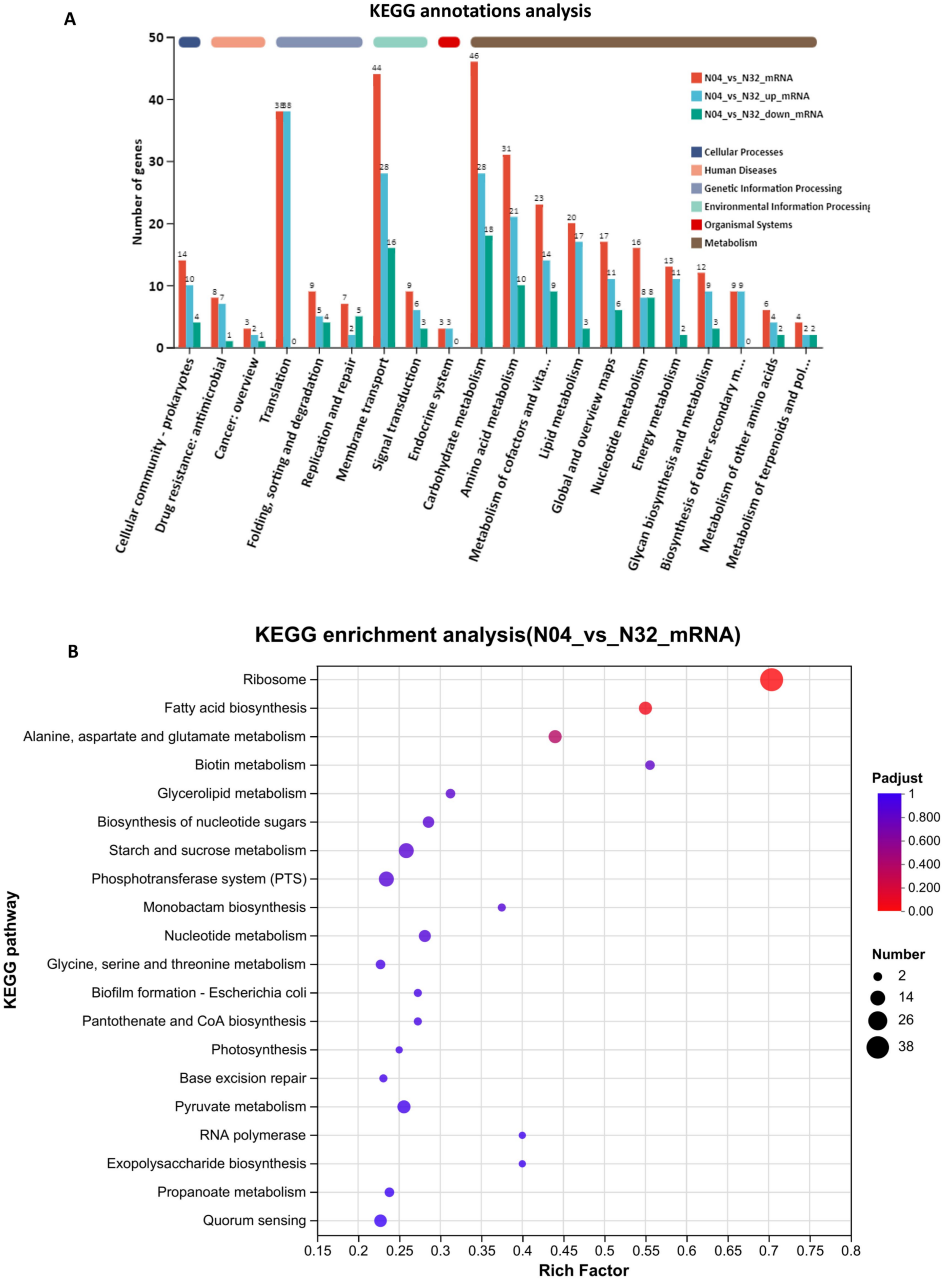
KEGG classification and enrichment of differentially expressed genes in N04 vs. N32. KEGG pathway annotation of DEGs between N04 and N32 **(A)**. KEGG pathway enrichment analysis of DEGs between N04 and N32 **(B)**. Each bubble represents a significantly enriched pathway, with the size corresponding to the number of DEGs and the color indicating the adjusted *p*-value.

The KEGG annotation bar chart supports this metabolic transition, demonstrating that the majority of DEGs are associated with metabolism-related categories, such as “carbohydrate metabolism” (46 genes) and “amino acid metabolism” (31 genes), which dominate the profile, whereas non-metabolic categories are considerably smaller (Figure 9A). KEGG pathway enrichment analysis of the N04 vs. N32 transcriptomes revealed a coordinated reprogramming of core metabolic and translational pathways, which was consistent with increased NMN production (Figure 9B). The KEGG enrichment analysis further uncovered significant metabolic pathways in N04, including those related to ribosomes, fatty acid biosynthesis, and alanine, aspartate, and glutamate metabolism. The notable enrichment of ribosome pathways is in an agreement with the results of the GO analysis, highlighting the essential role of ribosomes in NMN biosynthesis. The upregulation of fatty acid biosynthesis may reflect increased synthesis of NADPH, a key cofactor for NMN assembly, which is facilitated by the reductive power generated through acetyl-CoA carboxylase activity. Xiong et al. (2025) demonstrated that engineering the NMN biosynthesis-related pathway led to a 73% increase in NADPH levels and redirected carbon flux toward NMN biosynthesis, thereby enhancing precursor availability for nucleotide assembly. Furthermore, the enrichment of alanine, aspartate, and glutamate metabolism suggests a metabolic shift toward the production of aspartate-derived precursors for the NAD^+^ salvage pathway, directly linking amino acid flux to NMN yield. Collectively, these findings demonstrate that *lp2514* overexpression significantly alters the transcriptome of *L. plantarum*, optimizing metabolic pathways associated with NMN biosynthesis. This research provides a scientific basis for employing *L. plantarum* as a host for NMN production and lays the groundwork for future industrial applications.

### 3.6 Validation of DEG expression levels via RT-qPCR

To validate the reliability of the RNA-seq data, a subset of DEGs (upregulated, downregulated) identified between N04 and N32 was selected for RT-qPCR analysis (Table 2). Genes were chosen on the basis of their fold change values, involvement in NAD^+^ metabolism, and relevance to membrane transport processes as indicated by GO and KEGG enrichment analyses. The RT-qPCR results were strongly concordant with the RNA-Seq data (Spearman’s *r* = 0.93, *p* < 0.0001), confirming the upregulation of key transport-related genes and NAD^+^ biosynthesis genes in the N04 strain (Table 4). Notably, *lp3556* (encoding L-ribulokinase) exhibited a substantial upregulation (RNA-seq = 3.47; RT-qPCR = 3.66), suggesting a shift toward ribulose metabolism potentially linked to enhanced precursor availability for NMN biosynthesis. The key upregulated genes included ribosomal subunits (*lp1032*, RNA-seq = 1.61; RT-qPCR = 1.89) and NAD^+^ salvage enzymes (*lp2830* = 2.71; RT-qPCR = 2.54), which directly support enhanced translational capacity and aspartate-derived precursor supply for NMN biosynthesis. Strikingly, *lp2703*, which is involved in aspartate carbamoyltransferase activity, was markedly downregulated (RNA-seq = −2.27; RT-qPCR = −1.74), possibly reflecting alterations in pyrimidine biosynthesis under *lp2514*-mediated conditions. Together, the RT-qPCR results were consistent with the RNA-seq data, confirming the transcriptional regulation of key metabolic nodes.

**Table 4:** Validation of DEGs in *lp2514*-overexpressing *L. plantarum* via RT-qPCR.

Gene ID	Description	Fold change (N04 vs. N32) Log_2_FC	
		RNA-seq	RT-qPCR
*lp1032*	30S ribosomal protein S10 (*rpsJ*)	1.61	1.89
*lp1047*	50S ribosomal protein L5 (*rplE*)	1.51	1.77
*lp1058*	Adenylate kinase (*adk*)	1.95	2.53
*lp2900*	Hypothetical membrane protein	3.73	4.21
*lp2710*	Xanthine permease	3.12	2.79
*lp2830*	Aspartate ammonia-lyase (*aspA*)	2.71	2.54
*lp2771*	Nicotinate phosphoribosyltransferase (*pncB*)	0.91	1.89
*lp3556*	l-ribulokinase (*araB*)	3.47	3.66
*lp3545*	d-arabitol-phosphate dehydrogenase (*gutB*)	1.44	1.52
*lp0230*	PTS system % 2C mannitol-specific EIICB component	−3.17	−2.54
*lp2302*	Nicotinamide-nucleotide amidase (*cinA*)	−1.05	−2.43
*lp3555*	L-ribulose 5-phosphate 4-epimerase (*araD*)	−2.44	−2.31
*lp2183*	ADP-ribose pyrophosphatase	−1.40	−2.50
*lp2703*	Aspartate carbamoyltransferase (*pyrB*)	−2.27	−1.74
*lp2251*	Ribokinase (*rbsK2*)	−1.47	−1.21

### 3.7 Characterization of *cinA* in NMN biosynthesis via CRISPR/Cas9

**Figure 10:**
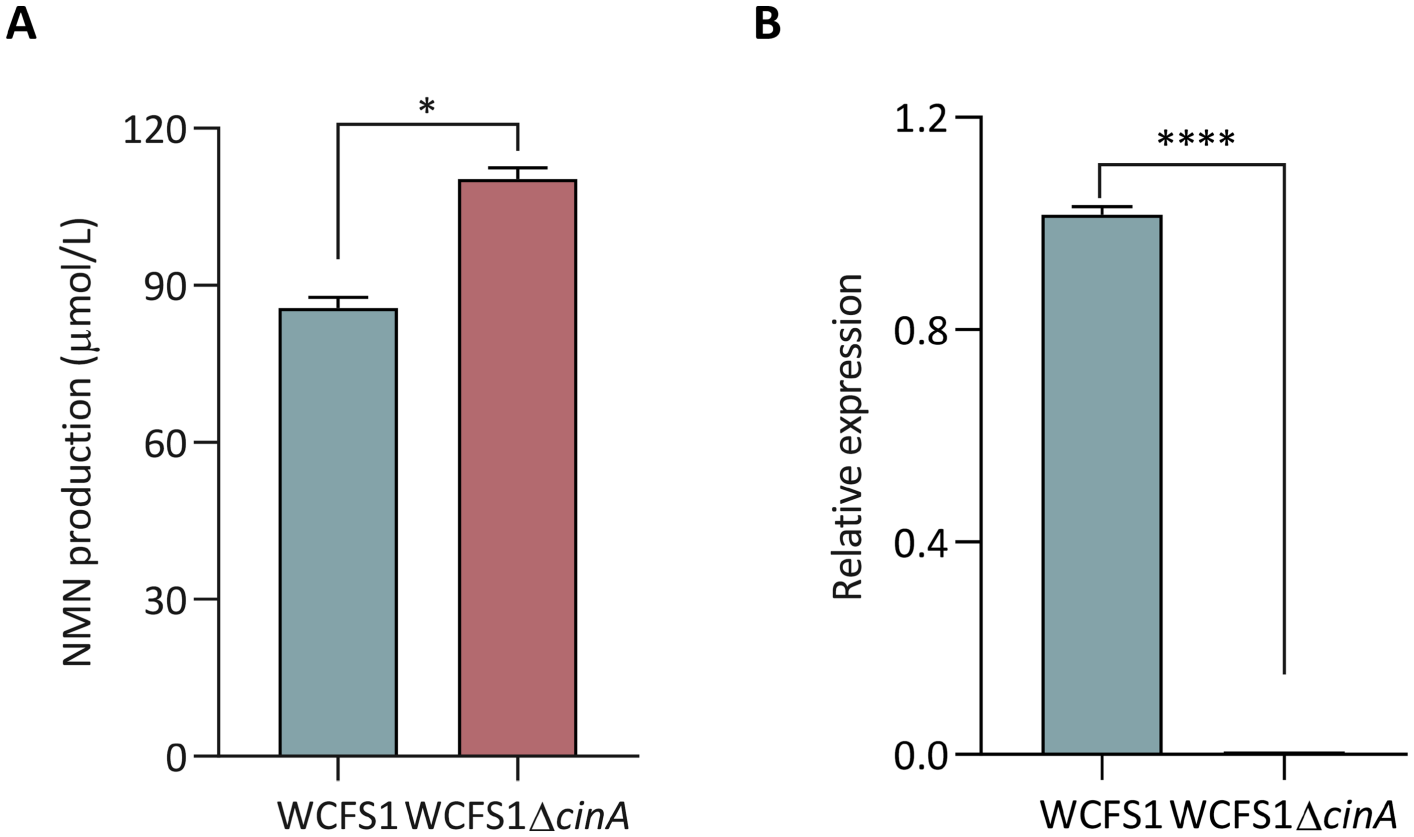
Characterization of the effect of *cinA* on NMN biosynthesis via CRISPR/Cas9 in *L. plantarum* WCFS1. Intracellular NMN production of the WCFS1Δ*cinA* mutant **(A)**. Analysis of the expression level of the WCFS1Δ*cinA* mutant by RT-qPCR **(B)**. *p < 0.05 , ****p < 0.00001.

To further validate the influence of DEGs on NMN biosynthesis, we targeted *cinA*, encoding nicotinamide-nucleotide amidase, which was downregulated in the *lp2514*-overexpressing strain and implicated in NMN turnover (Table 4). A *cinA* deletion mutant (WCFS1Δ*cinA*) was constructed (Supplementary Figures S3, S4) and assayed alongside wild-type WCFS1 for NMN production. As shown in Figure 10A, WCFS1Δ*cinA* accumulated significantly greater amounts of NMN than did the parental strain 29%, which was consistent with the removal of the enzyme responsible for NMN degradation. Zhang et al. (2024) similarly showed that deletion of the *cinA* in *Bacillus subtilis* enhanced NMN synthesis, consistent with our findings. RT-qPCR analysis (Figure 10B) revealed the absence of *the cinA* transcript in WCFS1Δ*cinA* and the upregulation of key salvage pathway genes (e.g., *aspA*) and ribosomal components (*rpsJ*), mirroring the global enhancements in precursor flux and translational capacity identified by RNA-seq. These data substantiate a dual mechanism-enhanced NAM uptake via *lp2514* and prevention of NMN hydrolysis through *cinA* deletion-underscoring the value of combining transporter engineering with targeted gene knockouts to maximize NMN biosynthesis. Together, these findings highlight the robustness of the sequencing data and suggest that *lp2514* overexpression, in conjunction with NAM supplementation, modulates central carbon and nucleotide metabolism in *L. plantarum*. Such regulatory shifts are likely instrumental in enhancing NMN biosynthetic potential, laying a molecular foundation for further metabolic engineering aimed at boosting NAD^+^ cofactor synthesis in *Lactobacillus*.

## 4 Conclusion

In this study, *lp2514* was identified as a novel endogenous NAM transporter in *L. plantarum* through molecular docking and CRISPR/Cas9 validation. Overexpressing *lp2514*, particularly using multicopy expression strategy, significantly enhanced NAM uptake and activated NMN biosynthesis. Transcriptomic analysis revealed broad metabolic reprogramming, including upregulation of ribosomal and NAD^+^ salvage pathway genes, supporting increased biosynthetic capacity. Furthermore, deletion of *cinA* further improved NMN yields and validated transcriptome-derived insights. These findings elucidate a new mechanism of NMN biosynthesis in LAB and establish *L. plantarum* as a promising food-grade chassis for sustainable, cost-effective NMN production. This work provides a foundation for engineering probiotics to develop functional foods enriched with bioactive NAD^+^ precursors.

## Data Availability

The original contributions presented in the study are included in the article/Supplementary material, further inquiries can be directed to the corresponding authors.
