# Supplementary material for: Transcriptome-guided engineering of a native niacin transporter in Lactiplantibacillus plantarum unveils metabolic rewiring for NMN biosynthesis
Source: Front Microbiol. 2025 Jul 14;16:1637666. doi: 10.3389/fmicb.2025.1637666 (PMC12301409; doi:10.3389/fmicb.2025.1637666)
Supplement: Supplementary file 1 [file Data_Sheet_1.docx]

**Supporting information**

**Transcriptome-Guided Engineering of a Native Niacin Transporter in *Lactiplantibacillus plantarum* Unveils Metabolic Rewiring for NMN Biosynthesis**


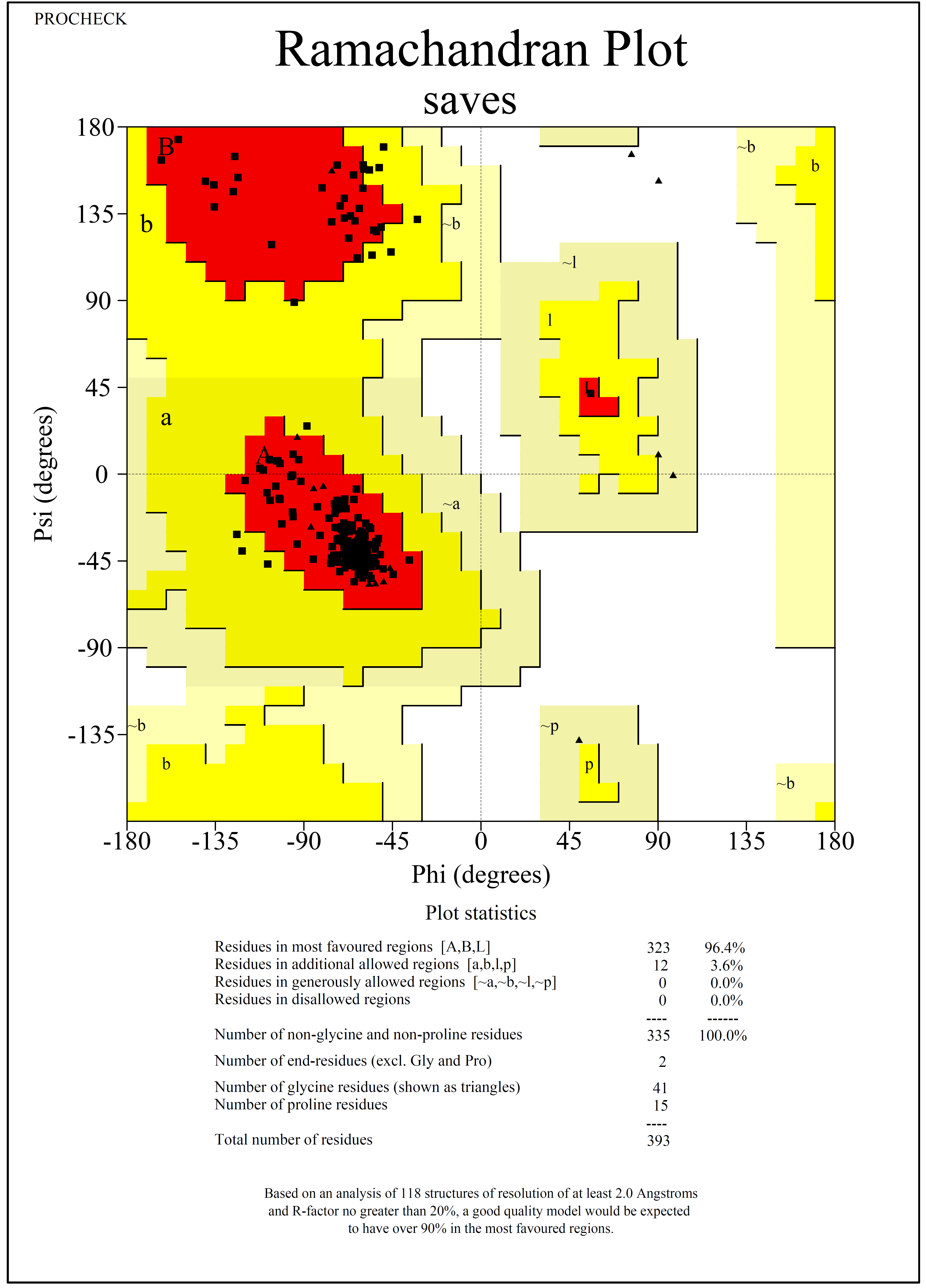


**Figure S1. Structural model of *lp2514* was generated by Ramachandran Plot.**
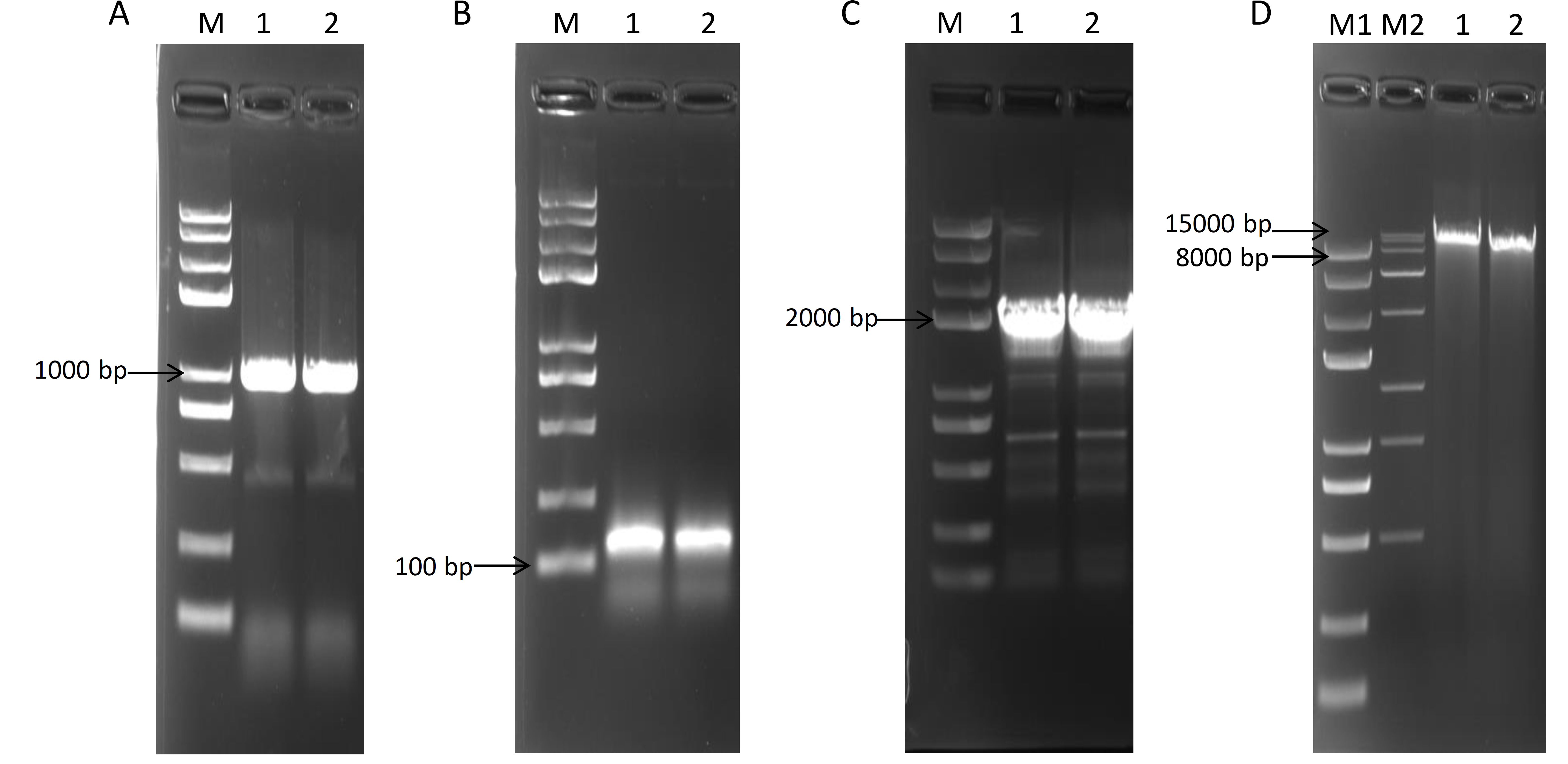


**Figure S2.** **Construction of the CRISPR/Cas9 plasmid of pLXY07.** (A) Amplification of the homology arm of target gene (*lp2514*) by PCR. M: 2K plus II marker; Line 1, 2: Homologous arms of *lp2514*. (B) Amplification of sgRNA of target gene (*lp2514*) by PCR. M: 2K plus II marker; Line1: sgRNA_*lp2514*; (C) Connection of the *lp2514* homologous arms and sgRNA_*lp2514* by overlap PCR. (D) Digestion of pLCP plasmid by *Apa* I and *Xba* I.

**Figure S3.** **Construction of the CRISPR/Cas9 plasmid of pHH13.** (A) Amplification of the homology arm of target gene and *sgRNA* (*cinA*) by PCR. M: 2K plus II marker; Line 1: *sgRNA_cinA*. 2, 3: Homologous arms of *cinA*. (B) Connection of the *cinA* homologous arms and sgRNA_*cinA* by overlap PCR. (C) Digestion of pLCP plasmid by *Apa* I and *Xba* I.

**Figure S4.** Characterization of *cinA* based on CRISPR/Cas9 in *L. plantarum* WCFS1. Identification of *cinA* deletion using colony PCR (A). The wild-type WCFS1 was used as control. The 2.1 and 3-kb bands indicating the positive and negative mutants, respectively. 2–9: transformants; 1: Wild-type. Sequencing validation from the WCFS1Δ*cinA* mutant (B).
